# Large-volume and deep brain imaging in rabbits and monkeys using COMPACT two-photon microscopy

**DOI:** 10.1038/s41598-022-20842-z

**Published:** 2022-10-22

**Authors:** Yuqing Lu, Xiangzan Wei, Wei Li, Xujun Wu, Chao Chen, Ge Li, Zhongqiang Huang, Yunfeng Li, Yu Zhang, Wen-Biao Gan

**Affiliations:** 1 School of Chemical Biology and Biotechnology, Peking University Shenzhen Graduate School, Shenzhen, 518055 China; 2 Institute of Neurological and Psychiatric Disorders, Shenzhen Bay Laboratory, Shenzhen, 518132 China; 3 School of Pharmaceutical Sciences (Shenzhen), Sun Yat-Sen University, Shenzhen, 518107 China; 4 Department of Orthopaedics, Peking 301 Hospital, Beijing, 100853 China; 5 Division of Hand and Microvascular Surgery, Department of Orthopedic Surgery, Shenzhen People′s Hospital, Shenzhen, 518020 China; 6 Guangdong Provincial Key Laboratory of Laboratory Animals, Guangdong Laboratory Animals Monitoring Institute, Guangzhou, 510663 China

**Keywords:** Neuroscience, Physiology, Optics and photonics

## Abstract

In vivo imaging has been widely used for investigating the structure and function of neurons typically located within ~ 800 μm below the cortical surface. Due to light scattering and absorption, it has been difficult to perform in-vivo imaging of neurons in deep cortical and subcortical regions of large animals with two-photon microscopy. Here, we combined a thin-wall quartz capillary with a GRIN lens attached to a prism for large-volume structural and calcium imaging of neurons located 2 mm below the surface of rabbit and monkey brains. The field of view was greatly expanded by rotating and changing the depth of the imaging probe inside a quartz capillary. Calcium imaging of layer 5/6 neurons in the rabbit motor cortex revealed differential activity of these neurons between quiet wakefulness and slow wave sleep. The method described here provides an important tool for studying the structure and function of neurons located deep in the brains of large animals.

## Introduction

In vivo imaging has become an important tool for understanding the structure and function of neurons and glial cells in the living brain^1–5^. Two-photon microscopy has been widely used for imaging cells located hundreds of micrometers deep below the surface of the rodent cortex^6–10^. Two-photon imaging has also been applied for studying neurons in the superficial cortical layers in non-human primates^11–14^. More recently, three-photon microscopy is developed for in vivo high-resolution imaging of hippocampus up to 1.1 mm below the cortical surface^15–17^. For imaging neurons located millimeters under the cortical surface, GRIN lens (gradient index lens) is typically the method of choice, as deep tissue imaging can be achieved with the inserted GRIN lens while maintaining synaptic resolution to study neuronal structure and function^18,19^. A recent study has used the GRIN lens for studying neurons in the monkey cortex^20^. One major limitation of imaging with GRIN lens, however, is a small field of view, which restricted the number of cells to be investigated. The larger field of view could be achieved with a larger GRIN lens, but at the cost of increased brain tissue damage.

By using a GRIN lens attached to a prism in combination with a thin-walled quartz capillary, an imaging method termed COMPACT (clear optically matched panoramic access channel technique) has been developed for deep and large-volume imaging of neuronal structure and activity in the mouse brain^21^. As the inserted GRIN lens could be rotated and moved up and down within the capillary, the COMPACT provides 360-degree panorama around the inserted probe and at different depths of the brain. Compared to the direct insertion of a GRIN lens into the brain tissue, the COMPACT can increase imaging volume by 2 to 3 orders of magnitude for the same volume of the inserted device. This COMPACT technique has been recently applied for imaging the mouse brain, but its application for imaging the brain of large animals has not been demonstrated.

In this study, we modified the COMPACT method for imaging neurons in living rabbit and monkey brains. We demonstrated that this method allowed for large-volume imaging of neurons located 2 mm below the surface of rabbit and monkey brains. With this approach, we found that somatic calcium activities of layer 5 or 6 neurons differed between SWS (slow wave sleep) and quiet wakefulness in rabbit motor cortex. Thus, the modified COMPACT technique in our study provides an important method for deep and large-volume imaging of neurons in large animals.

## Results

### Modification of COMPACT for large-volume deep brain imaging in large animals

**Figure 1:**
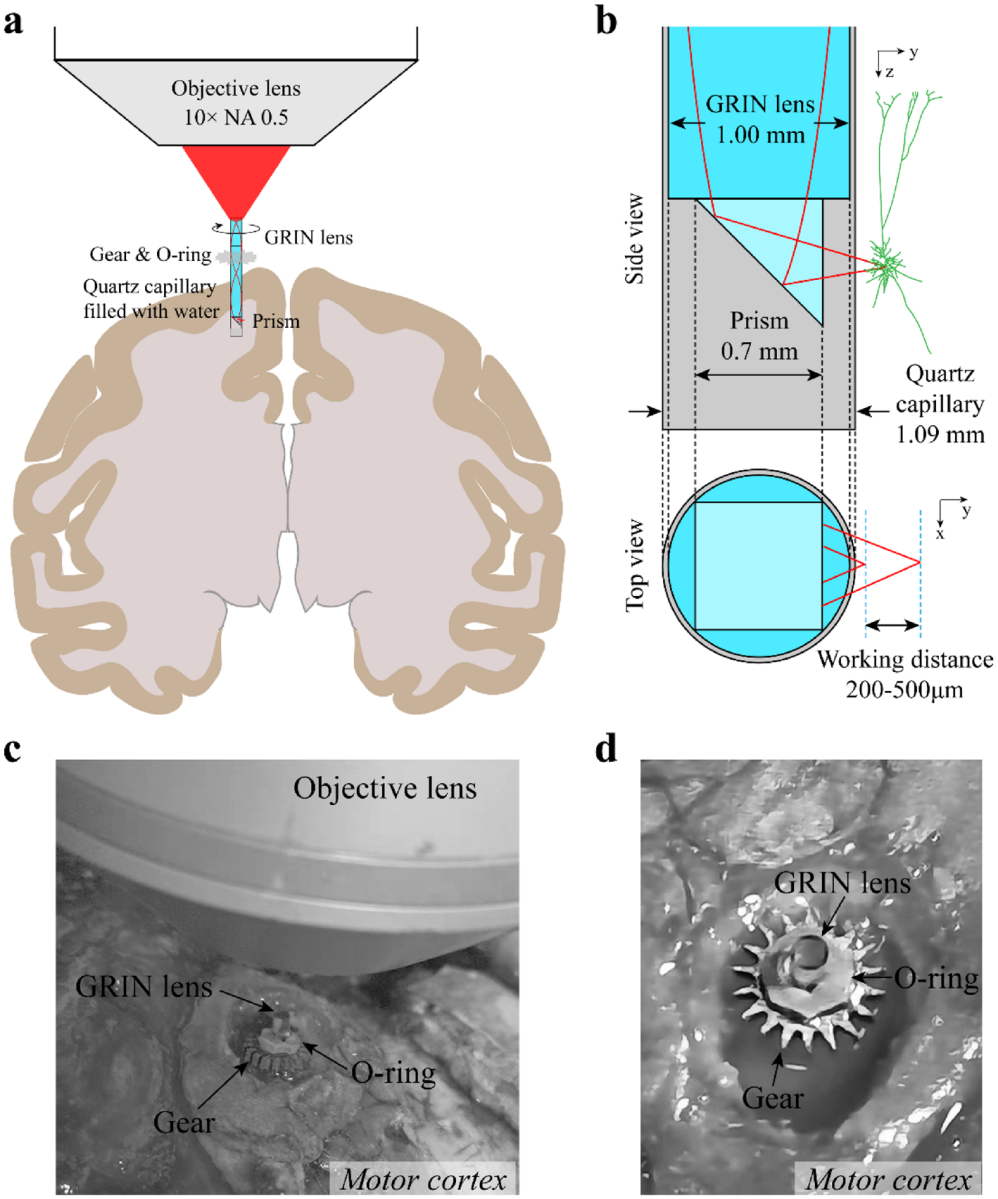
Modified design of COMPACT in large animal. (**a**) Schematic diagram of rotation and translation control for panoramic imaging at different depths. The gear rotates the imaging probe for the panoramic view, and the O-ring surrounds the imaging probe to move the capillary up and down for imaging at different depths in the brain. (**b**) Dimensions of the capillary, imaging probe and the working distance. The imaging probe consists of a 0.5 mm GRIN lens attached to a 0.35 mm prism, or consists of a 1.0 mm GRIN lens attached to a 0.70 mm prism. (**c, d**) Magnified views of the imaging probe, metal gear and O-ring. The schematic diagrams in (**a**) and (**b**) were created using Adobe Illustrator software, version 22.1 (https://www.adobe.com/products/illustrator.html).

For large-volume and deep brain imaging in large animals, we modified the recently-developed COMPACT technique used for imaging mouse brains^21^. We first implanted a thin-walled capillary into the brain of large animals as an optical channel, and then inserted the imaging probe composed of a GRIN lens and a prism into the capillary (Figs. 1a,b and S1). In order to rotate and move the imaging probe up and down within the capillary, we attached a metal gear and an O-ring to the imaging probe (Fig. 1a,c,d). The O-ring was made of rubber and could move along the surface of GRIN lens to change the depth of the imaging probe. The friction between the O-ring and GRIN lens was sufficient enough to keep the imaging probe at a certain depth. The metal gear had 18 teeth for controlling the rotation angle of the imaging probe. The gear-O-ring device reduced the volume of the original lens holder in mice, and was suitable for imaging in large animals.

In addition to the gear and O-ring, we also devised the fixation devices to fix the animal's head and body for stable imaging (Figs. S2,3). The head fixing frame was connected to the skull through the three-leg and four-leg head-posts (Fig. S2a-d). The fixation frame was linked to these head-posts with connectors which angles and directions could be adjusted, providing multiple force points to reduce the movement of the animal's head (Fig. S2e, f).

### Imaging structure and calcium activity of neurons in deep brain regions of rabbits

**Figure 2:**
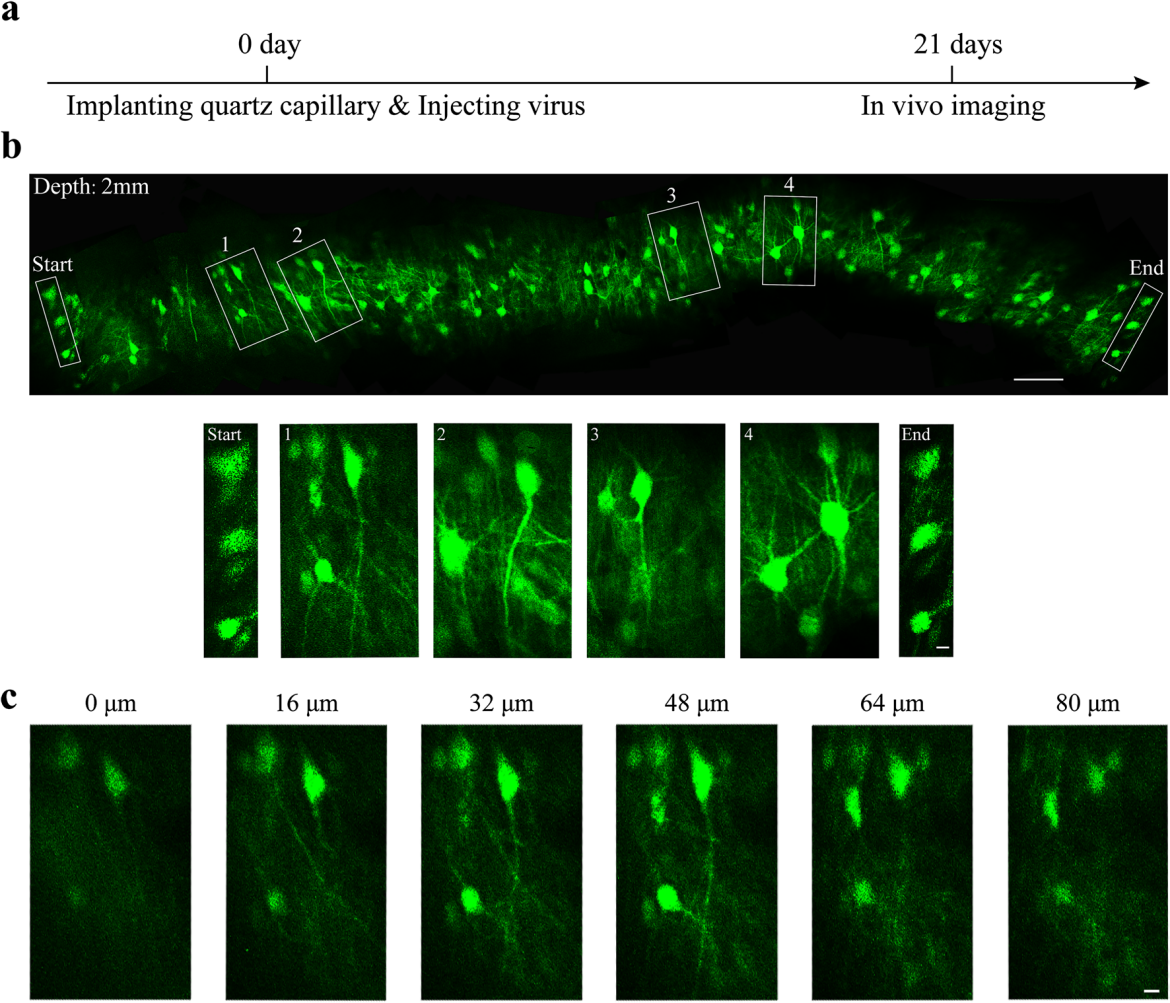
In vivo structural imaging of GFP-labeled neurons at the depth of ~ 2 mm below the surface of the rabbit brain. (**a**) The timeline for capillary implantation, AAV injection and in vivo imaging. (**b**) The top row represents 360° panoramic imaging of GFP-labeled neurons at the depth of 2 mm in the rabbit brain. The bottom row shows the magnified views of the boxed areas in the top row. “Start” and “End” indicate starting and ending points of the panoramic imaging. The images shown are the composite from one or two imaging sections. The scale bars in the top and bottom row are 100 μm and 10 μm respectively. (**c**) Z stack images of the box 1 in (**b**). The position where the neurons can be imaged is set as 0 μm, and the images are selected at the interval of 16 μm. Scale bar, 10 μm.

**Figure 3:**
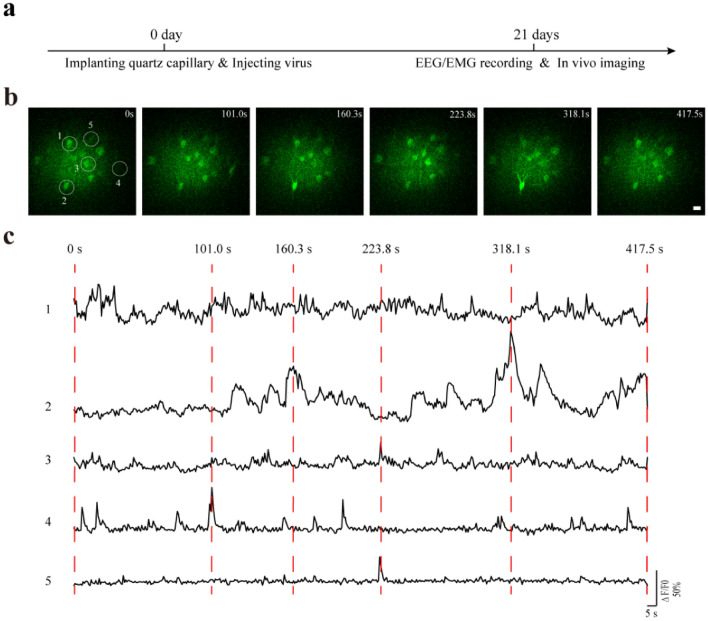
In vivo calcium imaging of GCaMP6s-labeled neurons in layer 5 of the rabbit motor cortex during quiet wakefulness. (**a**) The timeline for implantation, AAV injection and in vivo imaging. (**b**) Representative time-lapse imaging of neuronal calcium activity. The upper right corner shows the corresponding time point when an image was acquired. Scale bar, 20 μm. (**c**) Change of GCaMP6 fluorescence signals in somas circled in (**b**) during the quiet awake state.

To evaluate the capability of the modified COMPACT technique for in vivo imaging in large animals, we imaged structure and calcium activity of neurons located ~ 2 mm below the surface of rabbit motor cortex (Figs. 2, 3). We first surgically implanted quartz capillary, and injected AAV (adeno-associated virus) vectors expressing GFP (green fluorescent protein) or GCaMP6s. Approximately 20 days after the surgery and AAV injection, the second surgery was performed to implant head-posts and EEG/EMG (electroencephalogram/electromyogram) electrodes. Imaging was performed one day after the second surgery (Fig. 2a; see the method for details). By rotating the 0.5-mm-diameter probe over 360 degrees, we could obtain a ~ 2 mm-wide panoramic views of GFP-labeled neuronal structure with subcellular resolution (Fig. 2b,c). We also performed calcium imaging in layer 5 neurons expressing GCaMP6s during the quiet wake state, and detected changes of somatic calcium levels with high signal-to-noise ratios (Figs. 3 and S4). Thus, with the modified COMPACT technique, we were able to perform structural and calcium imaging of neurons in deep brain regions of rabbits.

### Imaging structure and calcium activity of neurons in deep brain regions of a monkey

**Figure 4:**
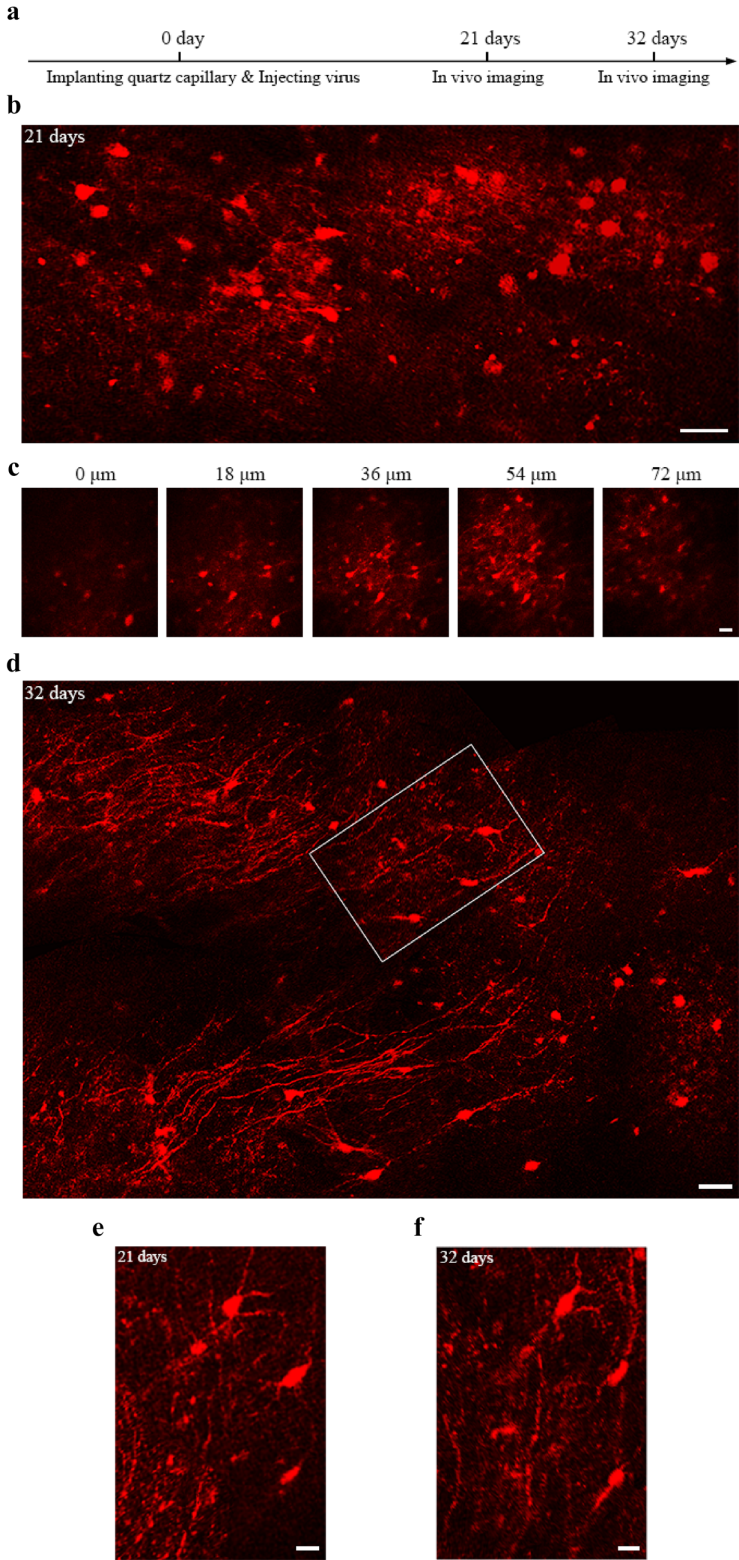
In vivo structural imaging of td-Tomato labeled neurons in anaesthetized monkey brain. (**a**) The timeline for capillary implantation, AAV injection and in vivo imaging. (**b)** Large-volume structural imaging of neurons at the depth of ~ 2 mm in the monkey brain 21 days after virus injection. The image shown is the composite from multiple image stacks. Scale bar, 50 μm. (**c**) A 3-dimentional image stack taken from a slightly different depth from **b** (~ 2.0 mm). The position where the neurons can be imaged is set as 0 μm, and images are selected at the interval of 18 μm. Scale bar, 30 μm. (**d**) Large-volume structural imaging of neurons at the depth ranging from 1.5 to 2 mm in the monkey brain 32 days after virus injection. The image shown is a composite. Scale bar, 50 μm. (**e, f**) Repeated imaging of the boxed area in (**d**) on the 21st and 32nd days after virus injection. The images shown are composite. Scale bars, 20 μm.

**Figure 5:**
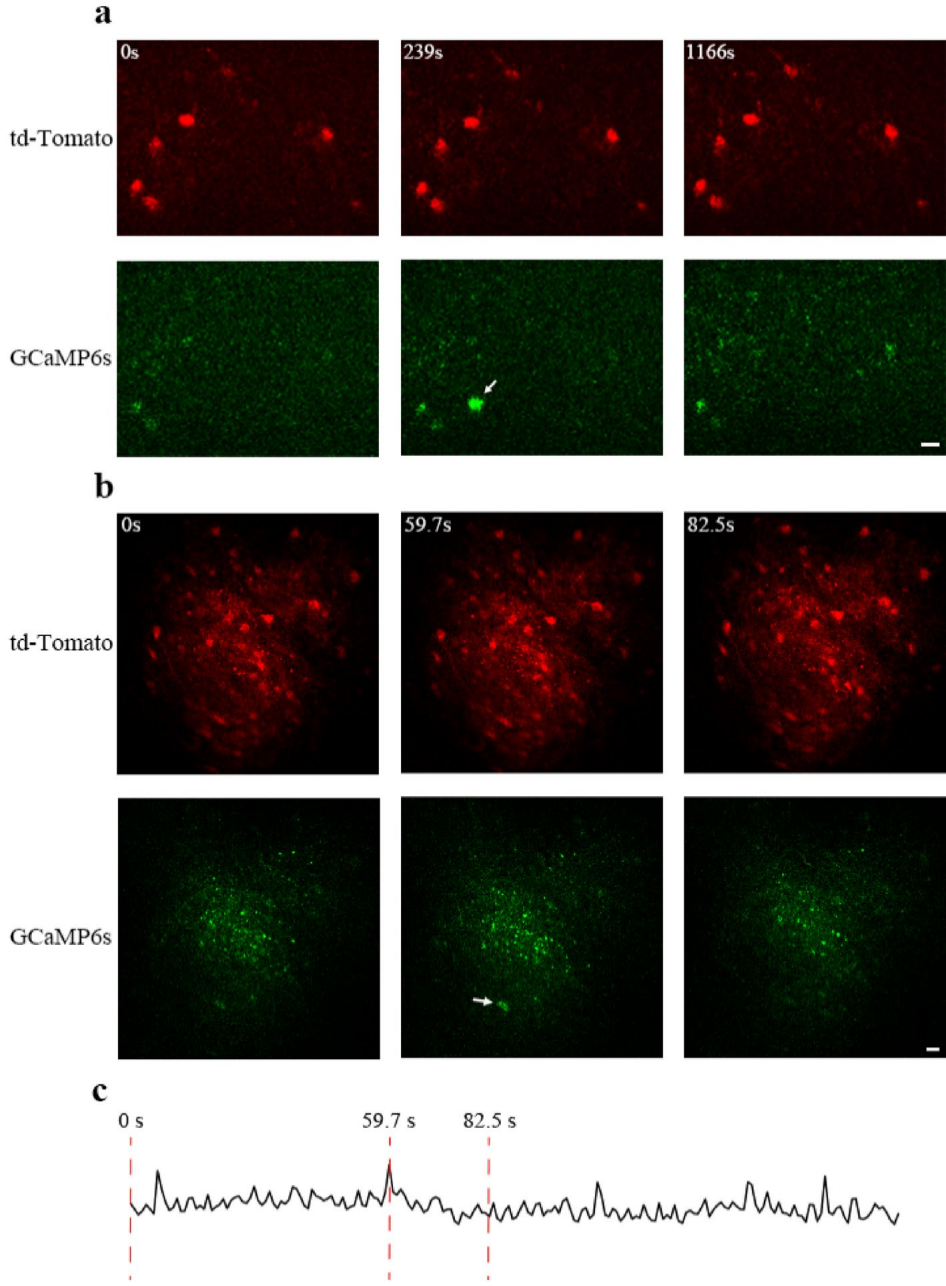
Detection of calcium signals in double-labeled neurons in the brain of an anesthetized monkey. (**a**) Representative structural and calcium images of neurons, which were double-labelled by td-Tomato (red) and GCaMP6s (green) 32 days after virus injection. The upper left corner of the image shows the time point when the images was taken. Increased GCaMP6s fluorescence was detected at 239 s as compared to other time points. Scale bar, 25 μm. (**b**) Representative time-lapse images of neuronal structure (red) and calcium activity (green) 32 days after virus injection. Increased GCaMP6s fluorescence was detected at 59.7 s as compared to other time points. Scale bar, 25 μm. (**c**) Change of GCaMP6 fluorescence signals in soma indicated by the arrow in (**b**).

To further evaluate the capability of the modified COMPACT technique for in vivo imaging of large animals, we imaged a monkey brain expressing td-Tomato and GCaMP6s in neurons located ~ 2.0 mm below the cortical surface (Figs. 4, 5). Similar to rabbit brain imaging, the surgical procedures for monkey brain imaging also involved two steps separated by 21 d. The first surgery included AAV injection and a quartz capillary insertion, and the second surgery only included the implantation of head-posts (Fig. 4a; see methods for details). Imaging was performed on the same day and ~ 11 d after the second surgery. Panoramic views of td-Tomato labeled neuronal structure in two regions were obtained in the anaesthetized monkey (Fig. 4b–d). The same neuronal structures previously imaged at 21 d could also be identified at 32 d (Fig. 4e,f). In addition, changes of somatic calcium activity could be detected in a few neurons in the isoflurane-anesthetized monkey (Fig. 5). Together, the results demonstrate that the modified COMPACT technique also allowed us to obtain large-volume and repeated structural imaging and calcium imaging in the deep brain regions of the isoflurane-anaesthetized monkey.

### Imaging calcium activity of layer 5/6 neurons during quiet wakefulness and slow wave sleep states

**Figure 6:**
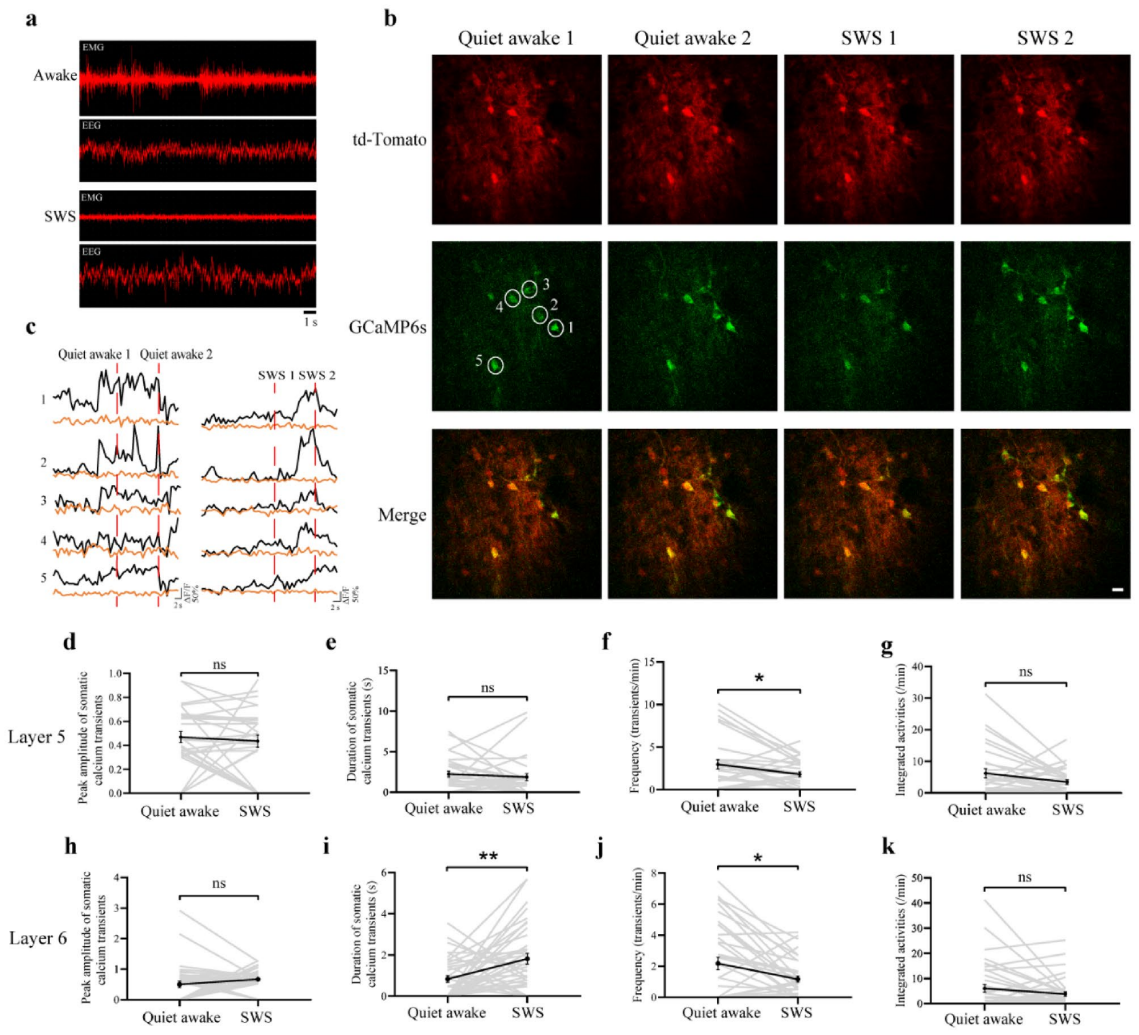
In vivo calcium imaging of layer 5/6 in the motor cortex of rabbits during quiet awake and sleep states. (**a**) Examples of the EEG and EMG traces for the identification of rabbit brain during quiet awake state and slow wave sleep (SWS). (**b**) Representative images of somatic structure (red) and calcium activity (green) in layer 5 neurons of the motor cortex during quiet awake state and sleep states. Scale bar, 20 μm. (**c**) Structure (Orange) and calcium (Black) fluorescence traces of somas circled in (b) during the quiet awake and sleep states in layer 5 of motor cortex. (**d-g**) The peak amplitude (**d**), duration (**e**), frequency (**f**) and total integrated activity (**g**) of somatic Ca^2+^ transients during quiet awake and slow wave sleep states in layer 5 of rabbit motor cortex (30 somas from 2 rabbits. Total of 29 quiet awake episodes and 28 SWS episodes. Quiet awake vs. SWS: *P* = 0.5833, 0.4761, 0.0145 and 0.0607 for amplitude, duration, frequency and total integrated activity, respectively). (**h–k**) The peak amplitude (**h**), duration (**i**), frequency (**j**) and total integrated activity (**k**) of somatic Ca^2+^ transients during quiet awake and slow wave sleep states in layer 6 of rabbit motor cortex (37 somas from 2 rabbits. Total of 43 quiet awake episodes and 43 SWS episodes. Quiet awake vs. SWS: *P* = 0.1486, 0.0035, 0.0101 and 0.1196 for amplitude, duration, frequency and total integrated activity, respectively). Data are presented as mean ± SEM.

Recent imaging studies in rodents have revealed differential neuronal activity in the superficial cortical layer between awake and sleep states, suggesting that activity-dependent processes differ between different behavioral states^22–25^. Using the modified COMPACT method, we imaged calcium activity of neurons in layers 5 and 6 of the rabbit motor cortex during quiet awake state and slow wave sleep (Fig. 6). There were no significant differences in amplitude, duration and total integrated calcium activity in layer 5 between the two states (Fig. 6d,e,g). We found that the frequency of somatic calcium activity in layer 5 was significantly higher in quiet awake state than in sleep (Fig. 6f). In addition, we found that the frequency of somatic calcium activity in layer 6 of the rabbit motor cortex was significantly higher in quiet awake state than in slow wave sleep (Fig. 6k), but the duration was lower in quiet awake state (Fig. 6j). There were no significant differences in total integrated calcium activity and amplitude of somatic calcium activity in layer 6 (Fig. 6h,i). These experiments further demonstrate the capability of the modified COMPACT technique for large animal brain imaging, revealing differential activity of layer 5/6 neurons in the rabbit cortex between different behavioral states.

## Discussion

Large animals, especially nonhuman primates, have become increasingly important in basic and clinical research, but in vivo imaging of neurons in deep brain regions of large animals remains difficult. In this study, we modified the recently-developed COMPACT technique and designed flexible head-fixed devices for large-volume structure and calcium imaging of neurons located 2 mm deep in the brains of head-fixed awake rabbits and an isoflurane-anaesthetized monkey. We further showed that neuronal calcium activity in layers 5 and 6 of the rabbit motor cortex differed between quiet wakefulness and slow wave sleep. The modified COMPACT technique described here provides an important method for studying deep brain structure and function in large animals.

Due to absorption and scattering of brain tissues, the imaging depth with two-photon microscopy is typically limited to ~ 600–800 μm. The imaging depth of three-photon microscopy improves to about 1 mm^15,17^. For brain tissues located several millimeters deep below the cortical surface, imaging could be performed with a GRIN lens, but with the limitation of a small field of view. The recently-developed COMPACT technique overcome such a limitation by implanting a thin-walled capillary into the mouse brain^21^, followed by inserting an imaging probe consisting of a GRIN lens and a prism inside the quartz capillary. The COMPACT technique can increase imaging volume by 2 to 3 orders of magnitude by rotating and moving the probe up and down, and has been used successfully for deep brain structure and calcium imaging in mice.

To use the COMPACT technique for large animal imaging, we designed a metal gear and a rubber O-ring to adjust the orientation and depth of the probe for large-volume imaging at a given depth in the brains of rabbits and monkeys (Figs. 2, 4). We designed the gear-O-ring instead of using the previously-described GRIN lens holder and electrical motor for controlling the imaging probe^21^ for the following two reasons: First, compared to the mouse head-fixation, large animal head fixation is more difficult and requires four-leg and three-leg head-posts. These head-posts occupy a large part of the animal's skull, and the region (the motor cortex) for implantation of capillary is close to the four-leg head-post. After placing the objective lens for brain imaging, it is difficult to fit in the GRIN lens holder attached to an electrical motor for controlling the imaging probe. The gear-O-ring is much smaller in volume than the previously-described lens holder and the electrical motor used in mice^21^, but allows for holding and rotating the imaging probe for large animal imaging. Second, compared to the previously-described GRIN lens holder with a minimum rotation interval of 1 degree, the minimum rotation interval of the gear-O-ring is 10 degrees. Although the accuracy is reduced with the gear-O-ring, it allows us to achieve stable and 360° panoramic imaging as shown in Figs. 1–6. This is because the gear-O-ring is tightly attached to the imaging probe and some overlap (~10 degree) between different fields of views is actually advantageous when rotating the imaging probe to obtain 360° panoramic imaging.

To minimize the damage to the brain caused by the implanted capillary, we used an imaging probe 0.5 mm in diameter in rabbits instead of the 1 mm probe used in mice^21^. In addition, the custom-designed head-fixing device could be adjusted to fit the heads of animals of different sizes (Figs. S2,3). These improvements allow us to achieve stable and repeated imaging of neuronal structure and calcium activity deep in the brains of large animals such as rabbits and monkeys. It is important to note that one limitation of the current COMPACT technique is its limited resolution with the numeric aperture ~ 0.4^21^, difficult to resolve postsynaptic dendritic spines. A two-photon endomicroscope has recently been developed to achieve deep-brain imaging at synaptic resolution by adding adaptive optics based on direct wavefront sensing^26^. Future improvement with a large GRIN lens and/or adaptive optics may help to increase the numeric aperture of the COMPACT technique for large volume and deep brain imaging at synaptic resolution.

With the modified COMPACT technique, we imaged calcium activity of neuronal somas in layers 5 and 6 of the rabbit motor cortex. The activity of neurons in layers 5 and 6 of the rabbit motor cortex differ during slow wave sleep from that during quiet wakefulness. Different patterns of neuronal calcium activity in the mouse cortex under different behavioral states are suggested to be involved in experience and sleep-dependent cortical plasticity^23–25^. It would be of interest to investigate whether and how awake and sleep-related neuronal activity regulates neuronal network plasticity and dynamics under physiological and pathological conditions in large animals.

## Methods

### Animals

The New Zealand rabbits were purchased from Guangdong Medical Experimental Animal Center and housed in the Animal Center of Shenzhen Institutes of Advanced Technology, Chinese Academy of Sciences and Shenzhen Research Institute of The Hong Kong Polytechnic University. Seven adult female rabbits (13–20 weeks of age and weighing 3.0–6.0 kg) were used in the study. The macaque (Macaca fascicularis) monkey was purchased and housed in the Institute of Guangdong Laboratory Animals. The adult male monkey used in the study was 7 years of age and weighed 6.0 kg. All experiments were approved by both Peking University's and Shenzhen Bay Laboratory's Institutional Animal Care and Use Committee (IACUC). All the experiments were conducted under the approved animal protocol in accordance with the institutional guidelines. All the experiments involving animals in the study are reported in accordance with the ARRIVE guidelines (PLoS Bio 8(6), e1000412, 2010).

### Surgical procedures for capillary implantation and adeno-associated virus (AAV) injection

The following surgical procedures were performed for implanting a thin-walled capillary into the brains of rabbits and monkeys (Fig. S1) and for injecting AAV viruses to label neurons with fluorescent indicators around the capillary:

First, animals (both rabbit and monkey) were anesthetized with 2.0~4.5% isoflurane, and the monkey was further anesthetized with additional Ketamine (10 mg/kg). The animal's head was fixed with a large animal stereotaxic apparatus (RWD Life Science Co., Ltd). The skin of the animal's head was cut to expose the skull region above the motor cortex in rabbits (identified according to the stereotaxic map of rabbits^27–32^, 3 mm anterior to bregma, 3 mm lateral from midline). The skull area over the motor cortex was then drilled with a miniature dental drill. A piece of skull ~ 3 mm in diameter was carefully removed with a pair of forceps, and the meninges overlying the cortex were further removed with a pair of fine forceps and scissors.

To implant the quartz capillary inside the brain, a computer-controlled electric controller (Z825B, Thorlabs) was used to slowly insert a thin-walled quartz capillary (Hilgenberg GmbH, 8 mm in length, 0.70/1.09 mm of outer diameter, 15 μm in thickness) into the brain at the speed of 10 μm/sec. The location with few blood vessels was chosen for capillary insertion to minimize the bleeding. After the capillary reached the desired depth, the brain tissue inside the capillary was removed with a thin needle connected to a vacuum pump. ~ 10 min after the tissue inside the capillary was removed, the capillary was moved out of the brain at the speed of 10 μm/sec, leaving a hole of ~ 0.70/1.09 mm in diameter in the brain. Subsequently, a new quartz capillary (Hyde entrepreneurship Biotechnology Co., Ltd. or Hilgenberg GmbH, 8 mm in length, 0.70/1.09 mm of outer diameter, 15 μm in thickness) with one end sealed with silicone (KN-300 N, KANGLIBANG) was pushed down into the hole at the speed of 10 μm/sec to the desired depth. Artificial cortical spinal fluid (ACSF) was used to flush the cortex if bleeding occurred.

Next, Adeno-associated virus (AAV) vectors encoding GFP (green fluorescent protein), td-Tomato, or genetically encoded calcium indicator GCaMP6s were injected near the capillary in the animal's brain. AAV9-hSyn-GFP (Titer: 4.63 × 10^13^ v.g./ml) purchased from WZ Biosciences was used to label neuronal structure in the rabbit brains. A mixture of three different viruses (AAV2/9-CAG-flex-GCaMP6s (Titer: 7.31 × 10^12^ v.g./ml): AAV2/9-hSyn-Cre (Titer: 2.28 × 10^13^ v.g./ml): AAV2/9-CAG-flex-td-Tomato (Titer: 7.44 × 10^12^ v.g./ml) = 10:1:1, OBiO) were used for co-labeling of neurons with td-Tomato and GCaMP6s in the rabbit and monkey brains. These viruses were injected through a sharp glass electrode attached to a pressure injection device (AONIEN; 30 p.s.i., 20 ms, 0.33 Hz). The precise locations and depths of the injection sites were controlled by an electrically controlled micromanipulator (Z825B, Thorlabs). We injected 0.1 µL of the virus at each location, ~ 10 locations per animal. The electrode remained for 5 min at the injection site after each injection to minimize the spread of the viruses to other areas. The spread area of the virus appeared to be limited and only a few cells were labeled near capillary along the radial direction (typically within the range of ~100 μm), within the working distance of the objective (200 μm-500 μm). When we imaged the monkey brain on day 21, we found that only few cells were labeled with td-Tomato and GCaMP6s. We further imaged the monkey brain again on day 32, when the number of td-Tomato-labeled cells appeared to increase and GCaMP6 fluorescence could be observed in few neurons.

After virus injection, silicone adhesive (Kwik-SIL, WPI) was used to seal the meninges, and dental cement (NISSIN) was used to fix the capillary to the skull. To prevent the capillary from dust and damage, we placed a metal protective cap around the capillary. Finally, we sutured the skin and applied Lincomycin Hydrochloride and Lidocaine Hydrochloride Gel (New Asia Pharmaceutical, C_18_H_34_N_2_O_6_S: C_14_H_22_N_2_O–HCl = 5:4) to the surgical location to avoid pain and infection.

During the three-week recovery period after the surgery, the rabbits were intramuscularly injected with ceftriaxone sodium (Youcare pharmaceutical group, 50 mg/kg) every two days, and the animals were able to engage in routine activities without affecting the implanted capillary.

### Surgery for imaging and EEG/EMG recording

Three weeks after AAV injection, the second surgery was performed to attach the head-posts for stable in vivo imaging and to implant four electrodes for EEG/EMG recording.

The four-leg and three-leg head-posts were attached to the animal head by stainless steel cranial screws (Fig. S2) as well as by the dental cement. According to the shape of the animal's skull, we first used the vise to slightly adjust the shape of the legs of the head-posts to make the head-posts fit better with the skull surface. After placing the head-posts over the skull, we then marked the positions of R5 holes on the skull and drilled holes at the marked positions. We then implanted stainless steel nails into the skull through the R5 holes until the three-leg and four-leg head-posts were firmly fixed to the skull. Finally, an appropriate amount of dental cement was used for further connecting the head-posts with the skull.

For EEG recording in rabbits, two holes (1.5 mm in diameter) were drilled with an electric mill above the visual cortex and cerebellum^33–36^. Two silver-plated screws (2 mm in diameter) connected to two epoxy-coated silver wire (0.008 inch (0.203 mm) in diameter, A-M Systems) were inserted into the two holes separately and were used as electrodes to record cortical EEG. One end of the screws touched the meninges, and the other end of the silver wire was soldered to a connector pin. Two electrodes for EMG recording made of insulated copper wire (300 μm in diameter) were placed on the nuchal muscle. Finally, all electrodes were stabilized with dental cement for stable recording of EEG and EMG signals.

### EEG/EMG recording and analysis

EEG/EMG was recorded using the BL-420F Biological Data Acquisition & Analysis System (Chengdu TME Technology Co., Ltd, China) with a bandpass setting of 0.5–100 Hz. Quiet awake state was identified by lower amplitude and higher frequency (> 10 Hz) EEG activity and medium-to-high muscle activity. Slow wave sleep was identified by higher amplitude and lower frequency (< 10 Hz) EEG activity and low muscle activity.

### Imaging probe bonding

The imaging probe was made of a high refractive index (N-LASF31, Joptics) right-angle micro-prism and a 0.5/1-mm-diameter GRIN lens (N.A. 0.5; GoFoton Nanjing Co., Ltd). To attach the GRIN lens with the microprism, one end of the GRIN lens was inserted into a sponge ~ 5 mm in thickness. A small amount of UV-curing optical adhesive (NOA81) was applied as the connecting glue between the GRIN lens and prism under a stereoscope. The right-angle microprism was placed in the center of the lens with a pair of forceps, and the optical adhesive was cured with UV light for 8 s. Before the use of the probe for brain imaging, the probe was first used to image 1-μm fluorescent beads in 1% agar to check the quality of the probe as described previously^21^ (Fig. S5).

### Inserting the GRIN lens into the capillary and aligning the objective with the GRIN lens

To align the imaging probe with the capillary and insert the imaging probe into the capillary, we first measured the depth required for the GRIN lens to enter the capillary. The gear-O-ring was then fixed to the corresponding position of the GRIN lens. Subsequently, we filled the glass capillary with water, used the plastic-tipped optical tweezers to clamp the top of the GRIN lens, aligned the GRIN lens with the capillary under the magnifying glass, and gently inserted the tip of the GRIN lens into the capillary. We then used the optical tweezers to gently turn the fixed gear on the GRIN lens such that the imaging probe would slowly slide along the capillary to the desired imaging position.

To align the objective with the GRIN lens, we first adjusted the level of the animal's head by rotating the Flat M4 × 14 in the head fixture (Fig. S2a) so that the surfaces of the GRIN lens and the objective lens was roughly parallel to each other. The following two steps were performed to further align the objective and GRIN lens. One was to move the head fixture along the X-Y directions to change the position of the GRIN lens to align with the objective lens horizontally, and the other was to rotate the objective on the two-photon microscope to align with the GRIN lens angularly. The alignment between the objective and GRIN lens was achieved when the surfaces of the objective and GRIN lens were parallel to each other and the excitation light from the objective was observed to pass through the GRIN lens.

### Structural and calcium imaging

Neuronal structure and somatic calcium imaging in rabbits was performed using a two-photon microscope (Bruker Ultima Investigator equipped with TI: Sapphire Laser) with the laser tuned to 920 nm and with a 20X air objective (N.A. 0.5). A 2 X digital zoom was used to yield images (295 × 295 μm, 512 × 512 pixels) for the quantification of neuronal structure (GFP/td-Tomato labeled) and calcium transients. With the Bruker's two-photon imaging system, the laser power and pixels dwell time for imaging were ~ 30 mw and 1.6 µs, respectively.

Neuronal structure and somatic calcium imaging in monkeys were performed using a Thorlabs Multiphoton Imaging Microscope (Bergamo II, Thorlabs Imaging Systems) with the laser tuned to 920 nm and with a 10X air objective (N.A. 0.5). A 3X digital zoom was used to yield images (443 × 443 μm, 256 × 256 pixels) for quantification of somatic structure (td-Tomato labeled) and calcium activity. With the Thorlabs' multiphoton imaging system, the laser power and pixels dwell time for imaging were ~ 30 mw and 5 µs, respectively.

### Calcium imaging data analysis

Changes in somatic calcium activity during quiet wakefulness and slow wave sleep were measured by changes in GCaMP6s fluorescence that were analyzed post hoc with ImageJ software (NIH) according to previous studies^37,38^. Regions of interests (ROIs) corresponding to visually identifiable somatic structure and calcium transients were selected for quantification. Changes of fluorescence ΔF/F_0_ was calculated as ΔF/F_0_ = (F − F_0_)/F_0_, in which F was measured by averaging pixels within each visually identifiable soma, and F_0_ was the average of 10% minimum F values during the imaging period. The threshold for detecting somatic calcium transients was > 3 S.D. of baseline fluorescence F_0_ for GCaMP6s. The peak amplitude was the maximum ΔF/F_0_ value of each calcium transient during the imaging period. Frequency of calcium transients was defined as the number of calcium transients per minute. Duration referred to the full width of each calcium transient above the threshold. The total integrated calcium activity was the sum of calcium activity above the threshold over 1 min.

### Statistics

All statistical analyses were performed using Prism 8.3.0 (GraphPad Software). All data are presented as mean ± SEM. Paired *t*-tests were used for comparison between different groups. *P* < 0.05 indicated the level of significant difference (*P < 0.05, **P < 0.01, ***P < 0.001).

## Supplementary Information

Supplementary Information.

## Data Availability

Data supporting the findings of this study are available from the corresponding authors upon reasonable request.
