# Supplementary material for: Large-volume and deep brain imaging in rabbits and monkeys using COMPACT two-photon microscopy
Source: Sci Rep. 2022 Oct 22;12:17736. doi: 10.1038/s41598-022-20842-z (PMC9588025; doi:10.1038/s41598-022-20842-z)
Supplement: Supplementary file 1 — Supplementary Information. [file 41598_2022_20842_MOESM1_ESM.pdf]

Supplementary Information

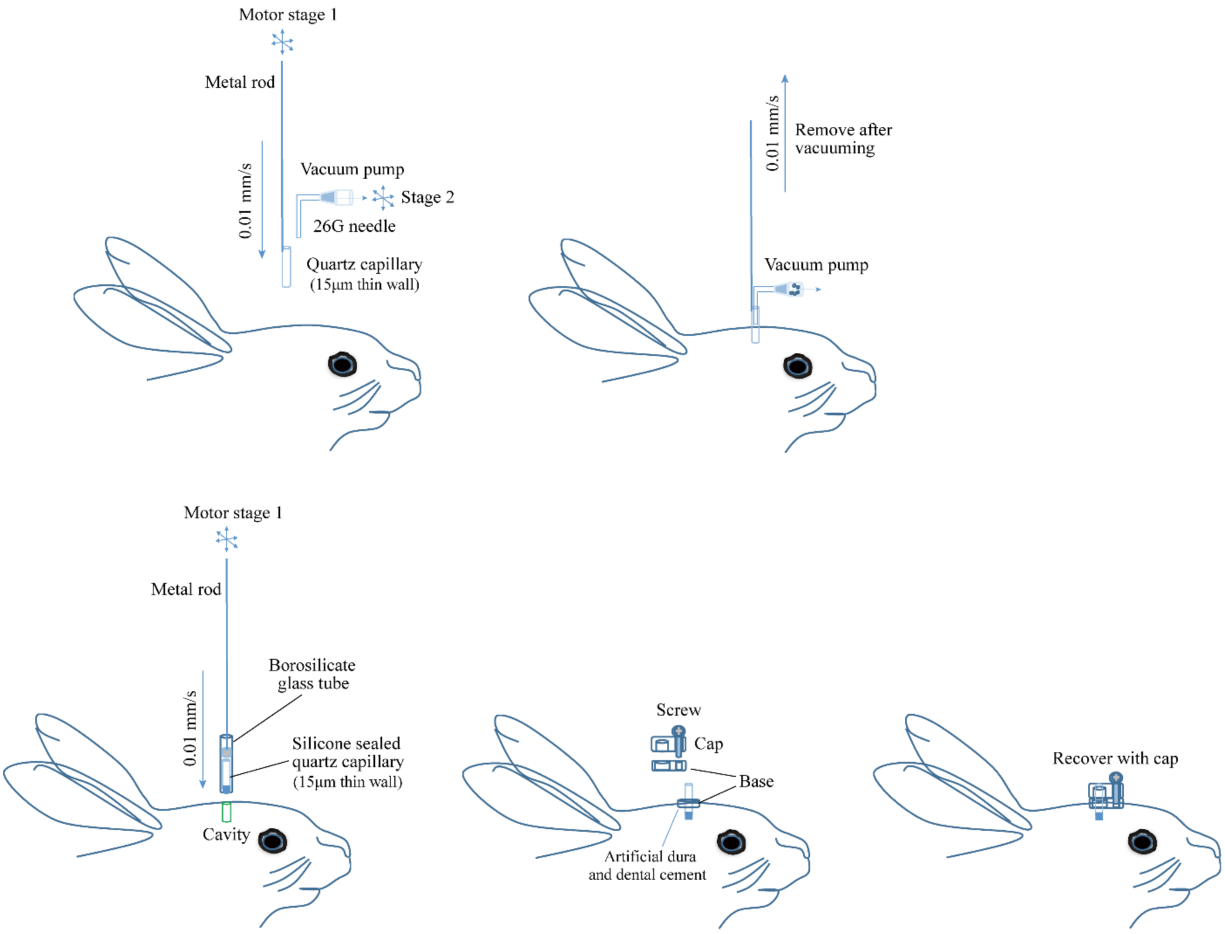

Supplementary Fig. 1 Surgical procedures for capillary implantation.

The schematic diagram was drawn using Microsoft PowerPoint 2021MSO, version 2207 Build 16.0.15427.20182 (https://www.microsoft.com/zh-cn/microsoft-365/buy/compare-all-microsoft-365-products).

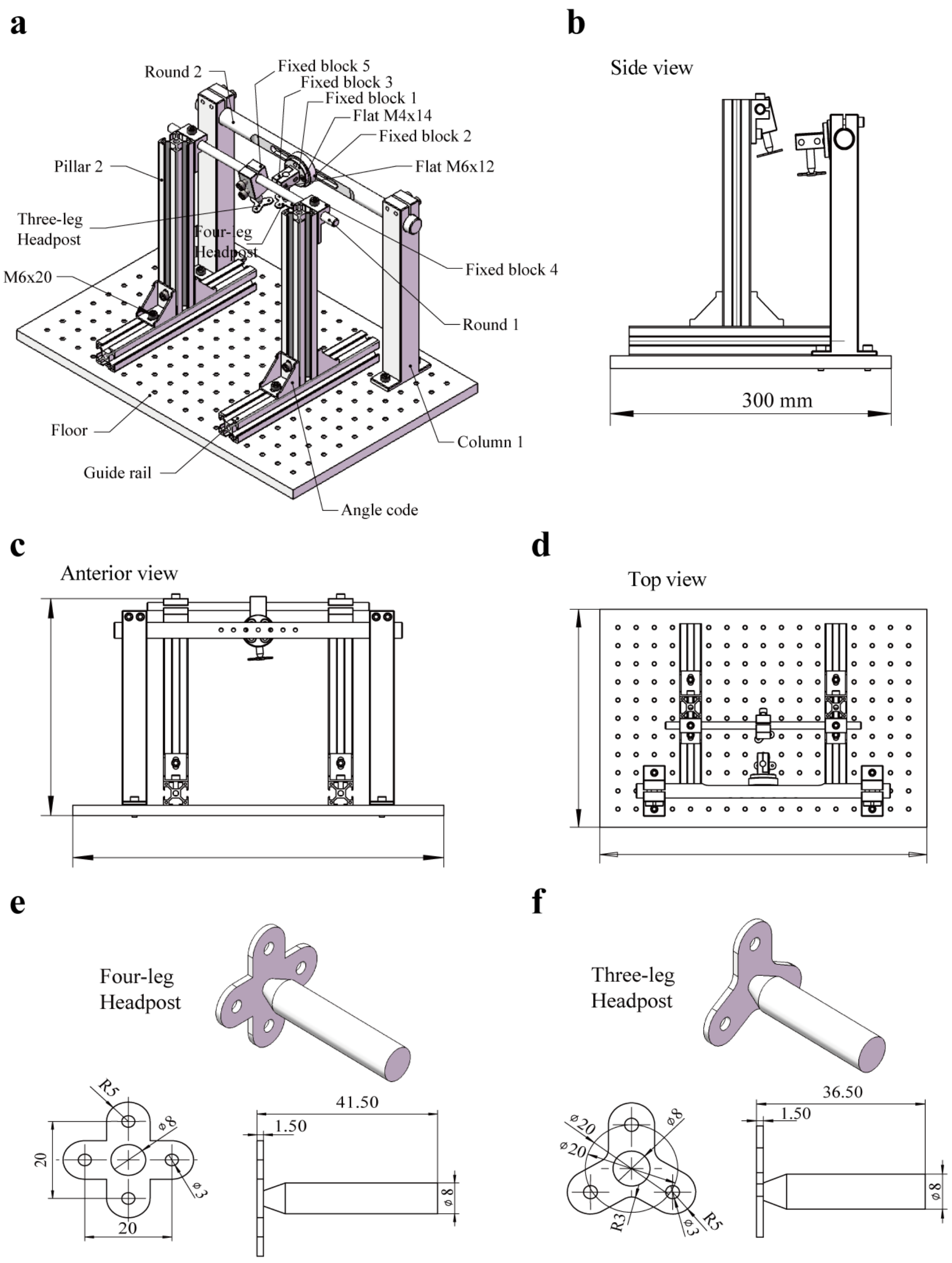

Supplementary Fig. 2 Fixation devices for fixing the animal's head.

(**a**) Head fixing device including seventeen parts, such as Grip-body, Fixed-plate and Square-block.

(**b-d**) The side view, anterior view and top view of head fixing device.

(**e**) Dimensions of four-leg head-post.

(**f**) Dimensions of three-leg head-post.

The schematic diagram was drawn using Autodesk AutoCAD 2014 software, version I.18.0.0 (https://www.autodesk.com.cn/products/autocad).

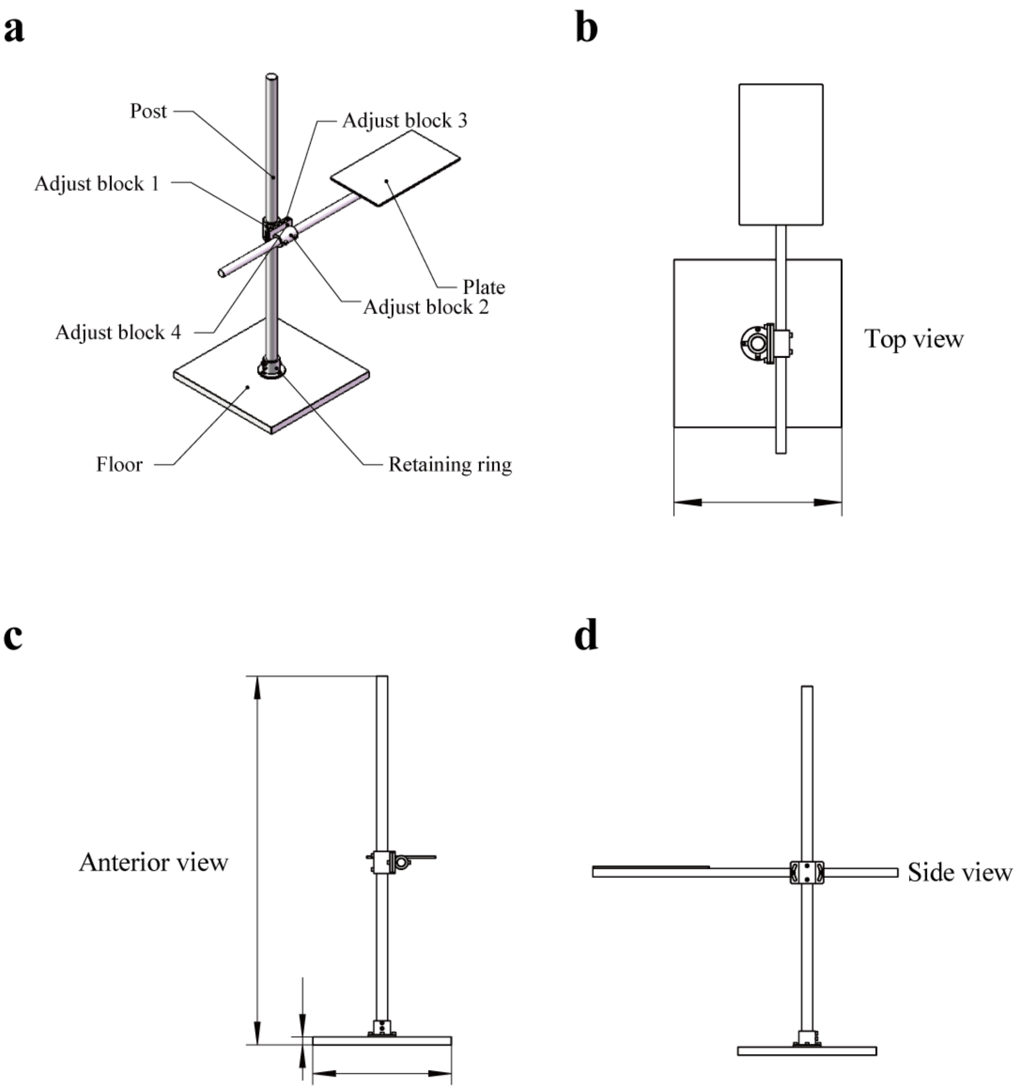

Supplementary Fig. 3 Fixation devices for fixing the animal's body.

(**a**) Body fixing device including nine parts, such as plate, adjust-block and post.

(**b-d**) The top view, anterior view and side view of body fixing device.

The schematic diagram was drawn using Autodesk AutoCAD 2014 software, version I.18.0.0 (https://www.autodesk.com.cn/products/autocad).

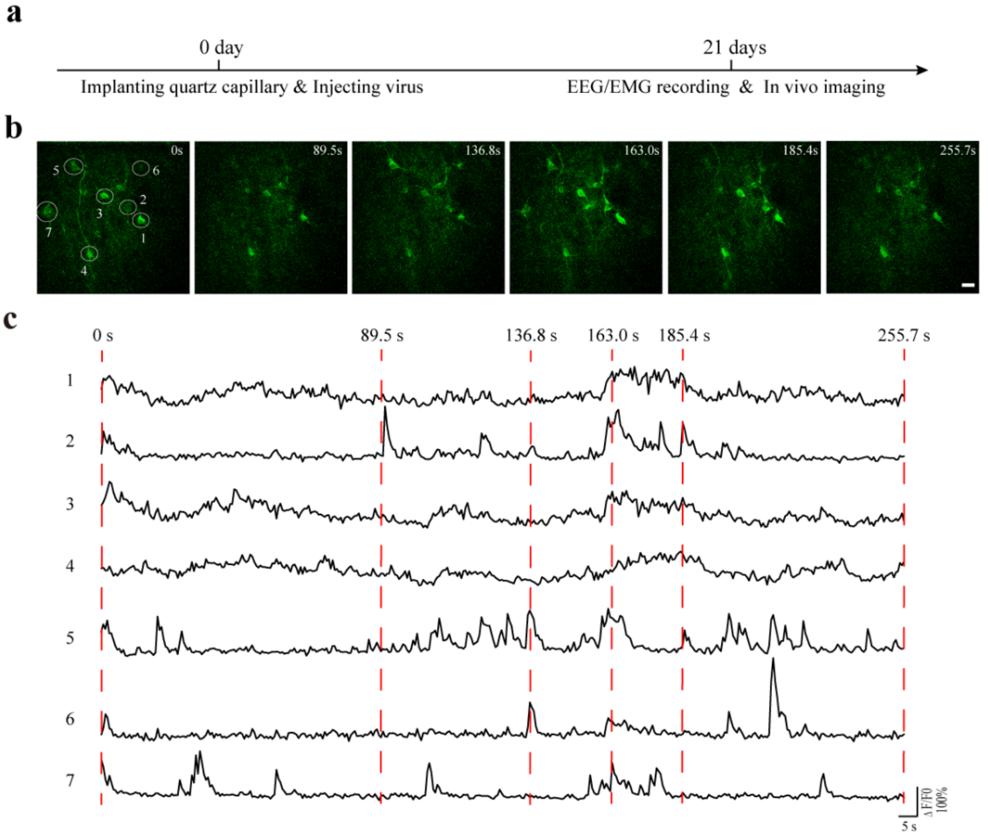

Supplementary Fig. 4 In vivo calcium imaging of GCaMP6s-labeled neurons in layer 5 of the rabbit motor cortex during quiet wakefulness.

(**a**) The timeline for implantation, virus injection and *in vivo* imaging. (**b**) Representative time-lapse imaging of neuronal calcium activity. The upper right corner of the image indicates the corresponding time point when the image was taken. Scale bar, 20 µm. (**c**) Calcium fluorescence traces of somas circled in (**b**) during the quiet awake state.

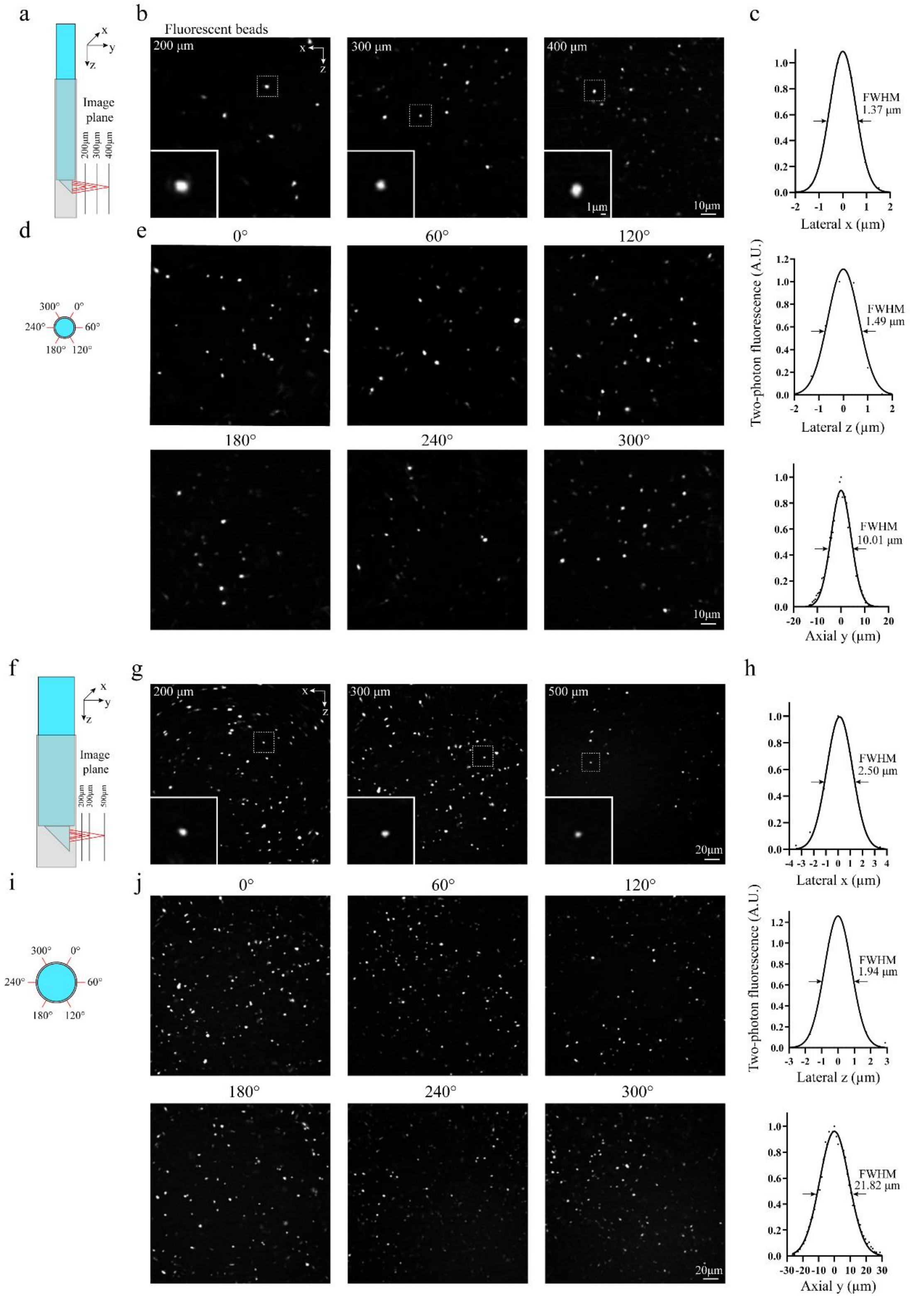

Supplementary Fig. 5 In vitro fluorescent beads imaging with different imaging probes.

(**a**) Illustration of imaging beads with an imaging probe composed of 0.5 mm GRIN lens and 0.35 mm prism at different distances from the prism surface. (**b**) Images of 1-

μm fluorescent beads at the distance specified in (**a**) from the capillary. (**c**) Cross-sectional views of the PSF (point spread function) at 300 μm outside the prism surface in three different axes: x, z, and y. AU, arbitrary unit; FWHM, full width at half maximum. (**d**) Illustration of imaging beads with an imaging probe composed of 0.5 mm GRIN lens and 0.35 mm prism at different angles. (**e**) Images of 1-μm fluorescent beads at the angles specified in (**d**) at a depth of 1.5 mm. (**f**) Illustration of imaging beads with an imaging probe composed of 1 mm GRIN lens and 0.7 mm prism at different distances from the prism surface. (**g**) Images of 1-μm fluorescent beads at the distance specified in (**f**) from the capillary. (**h**) The cross-section view of the PSF at 300 μm outside the prism surface in three different axes: x, z, and y. (**i**) Illustration of imaging beads with an imaging probe composed of 1 mm GRIN lens and 0.7 mm prism at different angles. (**j**) Images of 1-μm fluorescent beads at the angles specified in (**i**) at a depth of 2 mm.

The schematic diagrams in (**a**), (**d**), (**f**) and (**i**) were created using Adobe Illustrator software, version 22.1 (https://www.adobe.com/products/illustrator.html).
